# Pure shear model for crack width analysis of reinforced concrete members

**DOI:** 10.1038/s41598-023-41080-x

**Published:** 2023-08-24

**Authors:** Karolis Sakalauskas, Gintaris Kaklauskas

**Affiliations:** Department of Reinforced Concrete Structures and Geotechnical Engineering, Vilnius Gediminas Technical University, Vilnius, Lithuania

**Keywords:** Civil engineering, Environmental impact

## Abstract

Reinforcement corrosion in concrete structures with excessive crack width poses a high risk of reducing the structure's service life. The crack width behavior is one of the most complex aspects of the mechanics of reinforced concrete (RC). With most of the models used in practice being semi–empirical or empirical, very few analytical approaches have been proposed. However, the analytical models lack either accuracy or simplicity, or both. This paper presents a new analytical model, termed the Pure Shear Model, that predicts mean crack width by a simple formula. It is based on the partial interaction tension stiffening model considering a short RC tie subjected to short–term loading. The model assumes elastic material properties and neglects shrinkage, internal cracking, and slip at the interface. It presumes that the only deformations that occur in concrete are the shear strains due to shear lag that are taken constant across the cover thickness. Deplanation of concrete section due to shear lag results in crack width linearly increasing from zero at the bar to its maximum value on the surface of the RC member. Despite the simplicity of the proposed model, its accuracy in predicting mean crack width was shown to be comparable to that of the design code methods.

## Introduction

Concrete, after water, is the second most used material by humans. While concrete combined with reinforcement is the most successful structural material humans ever invented, it has shortcomings. Concrete industry is responsible for the 7% share of CO_2_ emissions. Concrete has a low tensile strength and is brittle. Due to low tensile strength, it cracks and, due to brittleness, it is not capable of resisting tensile stresses after cracking, what often leads to excessive cracking. Excessive crack width may cause aesthetic problems and give the impression that the structure is unsafe. More importantly, the corrosion of reinforcement due to moisture or other chemical materials getting into the cracks may lower the cross–sectional resistance and reduce the service life of the structure^1^. To avoid the aforementioned issues, design codes limit crack widths.

Crack width analysis is one of the most complex aspects of the mechanics of reinforced concrete. There is currently no complete, comprehensive, and accurate theory for predicting crack width. The vast majority of cracking models, including the design code methods (Eurocode^2^, Model Code 2010^3^, ACI 318–95^4^, ACI 318–99^5^), are empirical or semi–analytical and only a few models were purely analytical. In 1977, Leonhardt^6^ proposed one of the first analytical models. It was based on the stress–transfer approach developed by Saliger^7^ and the observations of Goto^8^ on internal cracking. The stress–transfer concept expresses the bar–concrete interaction via the bond stress–slip behavior. Noakowski^9,10^ proposed another analytical model by solving a differential bond–slip equation. In a similar way, following a closed–form solution by Balazs^11^ for the case of a single crack and using a bond–slip law from the Model Code 2010^3^, Debernardi and Taliano^12,13^ developed an analytical crack width model that took into account the bond damage in the zone close to a crack. While most of the analytical models were based on the stress–transfer approach, Beeby^14^ proposed an analytical model using an alternative concept, the no–slip theory, first introduced by Broms^15^. In this model, the single most important parameter controlling crack width was thickness of concrete cover.

The analytical crack width models generally lack accuracy and often are rather complex. A comprehensive statistical study of Lapi et al.^16^, including 380 experimental RC beams, has demonstrated that the most accurate are semi–analytical models, namely Oh and Kang^17^ Eurocode 2^2^, and Model Code 2010^3^. However, even these models have a 30% scatter which is considered to be large. Such a big scatter could be due to several reasons. The heterogenic structure of concrete, being responsible for the highly variable tensile strength and cracking characteristics, is to blame for a sizeable portion of the error. The crack behavior of RC ties differs from that of beams. A recent study by the authors^18^ has shown that the crack width behavior of small and large beams also significantly differs. The test results often lack consistency due to the missing information about the location of the recorded crack widths (the extreme tensile surface or the reinforcement centroid level, or the bottom layer of the reinforcement if it consists of several layers). Similarly, the models frequently do not specify the location where crack width is identified. As noted by Schlicke et al.^19^, an error is also introduced by the classical formula of crack width, $w$, obtained as a product of crack spacing, ${s}_{r}$, and the mean strain difference of reinforcement, ${\varepsilon }_{sm}$, and concrete, ${\varepsilon }_{cm}$:

1 $$w=({\varepsilon }_{sm}-{\varepsilon }_{cm}){s}_{r}$$

This formula assumes that crack width is constant across the cover depth. Experimental investigations on concrete crack width across the concrete cover were carried out by Husain and Ferguson^20^, Beeby^21^, Yannopoulos^22^, Tammo and Thelandersson^23^, Borosnyoi and Snobli^24^, and Naotunna et al.^25^. These tests have shown that for ribbed bars crack width at the surface of reinforcement was either negligible or significantly smaller than the width at the external surface of concrete. Borosnyoi and Snobli^24^ and Caldentey et al.^26^ have noted that the reduction of crack width at the bar surface is resulted by the presence of internal cracks forming at the ribs of the bar. Similar tests of RC ties with plain bars^25^ have demonstrated that the crack width at the bar interface was just slightly smaller than that on the concrete surface. Naotunna et al.^25^ have concluded that the specimens with ribbed bars behaved in a fashion more related to the no–slip theory, while specimens with smooth bars behaved in a manner more related to the bond–slip theory.

This paper proposes a new mean crack width model, termed the Pure Shear Model, being characterized as (1) fully analytical; (2) crack width expressed via a simple formula; (3) crack width predicted on the surface of an RC member. The model considers a short–term load. The proposed approach, in a simplified way, mimics the above tests^20–25^ and is based on a simple analogy to the deformation behavior of a short RC tie having the length of crack spacing. The accuracy of the proposed model is compared to the design code methods using an extended number of experimental RC ties.

## The basic idea: the mechanics behind the model

**Figure 1:**
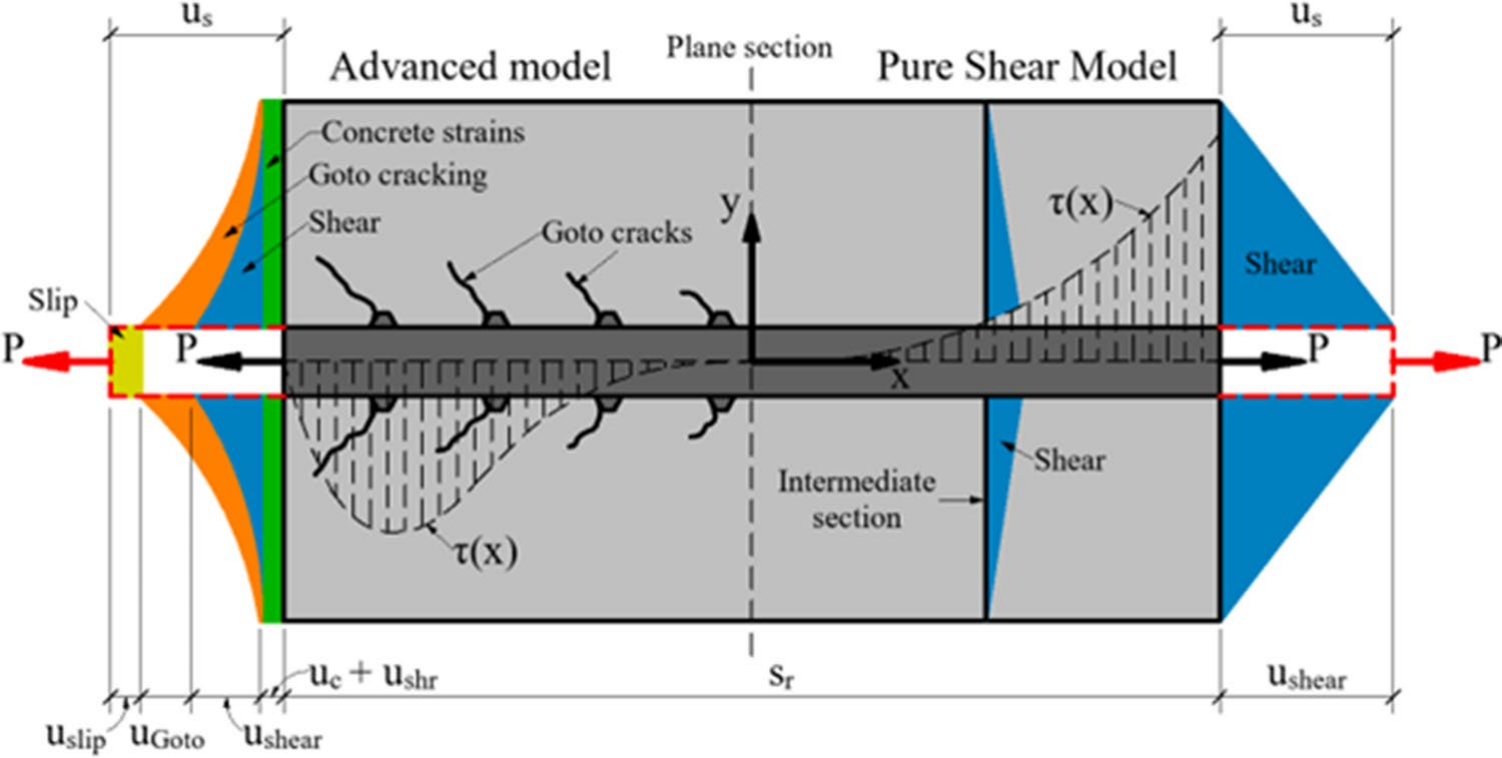
The Pure Shear Model.

The proposed crack width model was developed from the analogy to the deformation and cracking behavior of an RC tie reinforced with a single deformed bar and subjected to instantaneous axial force $P={{\varepsilon }_{si}{E}_{s}A}_{s}$, where ${\varepsilon }_{si}$ is the reinforcement strain at the point of application of external force, $P$, ${A}_{s}$ and ${E}_{s}$ are, respectively, the section area and modulus of elasticity of reinforcement. In accordance with Fig. 1, consider a single block of the RC tie representing the region between two adjacent cracks spaced at ${s}_{r}$. Presuming the block’s midpoint has a zero displacement, a longitudinal displacement of the reinforcement at the cracked section can be expressed via the sum of these displacement components as depicted in Fig. 1 (left):

2 $${u}_{s}\left(x\right)={u}_{c}\left(x\right)+{u}_{shr}\left(x\right)+{u}_{shear}\left(x\right)+{u}_{Goto}\left(x\right)+{u}_{slip}(x)$$

where ${u}_{c}(x)$ and ${u}_{shr}\left(x\right)$ are, respectively, the longitudinal displacements of concrete due to the external force, $P$, and shrinkage occurring prior to short–term loading;${u}_{shear}(x)$ is the shear displacement of concrete; ${u}_{Goto}(x)$ is the displacement due to the accumulated effect of Goto cracks^8^ forming at the ribs of the bar; ${u}_{slip}(x)$ is the slip of the reinforcing bar at the interface in respect to concrete.

In Eq. (2), the displacement components ${u}_{c}\left(x\right)$ and ${u}_{shr}\left(x\right)$ are related to the longitudinal strains of concrete. These components to some extent compensate each other as ${u}_{c}\left(x\right)$ is positive and ${u}_{shr}\left(x\right)$ is negative. In terms of absolute value, the latter parameter is considerably larger than the former. As the studies of Wu and Gilbert^27^ and Gilbert & Nejadi^28^ have demonstrated, even at first loading, drying shrinkage has a substantial effect on crack width. However, for simplicity reasons, the current model, similarly to many other models, will ignore the shrinkage effect. Displacement ${u}_{c}\left(x\right)$ will be also neglected.

Quantifying parameter ${u}_{Goto}\left(x\right)$, regardless of whether theoretical or experimental approaches are employed, is a significant challenge. As approximation, it can be assumed that ${u}_{Goto}$ is equivalent to the total crack width of the internal cracks forming within one–half of the RC block. For simplicity, the current model will assume the elastic behavior of concrete, thus parameter ${u}_{Goto}(x)$ will be neglected.

Slip ${u}_{slip}\left(x\right)$ (Fig. 1) can be related to crack width at the reinforcement. As discussed in the Introduction, the tests of Beeby^21^, Borosnyoi and Snobli^24^, and Naotunna et al.^25^ have demonstrated that the crack width at the immediate proximity to the reinforcement bar was rather small compared to the crack width on the surface. Therefore, for ribbed bars, the current model assumes ${u}_{slip}=0$.

Following the above and assuming that ${u}_{c}\left(x\right)={u}_{shr}\left(x\right)={u}_{shear}\left(x\right)={u}_{Goto}\left(x\right)={u}_{slip}\left(x\right)=0$, Eq. (2) gets the form:

3 $${u}_{s}\left(x\right)={u}_{shear}\left(x\right)$$

The displacement component ${u}_{shear}\left(x\right)$ exemplifies the shear lag effect. In the current context, this effect can be described as the deviation of concrete strains from the plane section due to shear (bond) stresses acting at the reinforcement–concrete interface. As seen in [Fig. 1 (right)], the shear displacement ${u}_{shear}\left(x\right)$ profile, similarly as bond stress, progressively increases from zero at $x=0$ to a maximum value at $x=\frac{{s}_{r}}{2}$. To further simplify the model, constant shear strain will be assumed across the concrete cover [Fig. 1 (right)].

Displacement ${u}_{s}\left(x\right)$ in a simple way can be related to crack width, $w$, defined on the surface of the member. Given the above simplifications [Fig. 1 (right)], for two adjacent blocks having length ${s}_{r}$, crack width $w$ can be expressed as

4 $$w={2u}_{s}\left(x=\frac{{s}_{r}}{2}\right)=2{u}_{shear}\left(x=\frac{{s}_{r}}{2}\right)$$

## Assumptions

The assumptions below reflect some of the above statements and introduce new hypotheses that will simplify the solution of Eq. (4).

1. All materials are elastic.
2. The stabilized cracking stage is considered: no new cracks will form.
3. Longitudinal concrete strains due to the external load and shrinkage are neglected.
4. Shear strains are taken constant across the concrete cover.
5. For ribbed bars, slip between reinforcement and concrete is neglected.

The collection of the aforementioned hypotheses makes the proposed approach best suited for predicting mean crack width.

According to assumptions 3 and 4, no other strains than pure shear strains act in the concrete. With assumptions 3, 4, and 5 introduced, shear lag is the only effect influencing crack width. Due to these assumptions, the proposed approach is named the Pure Shear Model. The next Section delivers a solution to Eq. (4).

## Derivation of the pure shear model

The current approach is based on the partial interaction tension stiffening model, which was explored in earlier studies^29–32^. Reinforcement displacement ${u}_{s}\left(x\right)$ can be related to strain ${\varepsilon }_{s}\left(x\right):$

5 $$ \frac{d{u}_{s}(x)}{dx}={\varepsilon }_{s}\left(x\right)\mathrm{ or }\frac{{d}^{2}{u}_{s}(x)}{d{x}^{2}}=\frac{d{\varepsilon }_{s}(x)}{dx}.$$

The below classical expression relates reinforcement strain to bond stress, $\tau (x)$ (e.g.,^33^):

6 $$\frac{d{\varepsilon }_{s}(x)}{dx}=\frac{4}{{E}_{s}{d}_{b}}\tau (x).$$

Combining Eqs. (5) and (6) yields to

7 $$\frac{{d}^{2}{u}_{s}(x)}{d{x}^{2}}=\frac{4}{{E}_{s}{d}_{b}}\tau (x).$$

Equation (7) has two unknowns. Based on assumption 4, the shear angle ${\gamma }_{c}\left(x\right)$ is expressed via the ratio of shear displacement ${u}_{shear}\left(x\right)$ to the concrete cover, $c$ (Fig. 1):

8 $${\gamma }_{c}\left(x\right)=\frac{{u}_{shear}(x)}{c}.$$

Using the above equation and assuming elastic material properties (Assumption 1), bond stress can be expressed via Hooke’s law for shear:

9 $$\tau \left(x\right)={\gamma }_{c}\left(x\right){G}_{c}=\frac{{u}_{shear}(x)}{c}{G}_{c}=\frac{{u}_{s}(x)}{c}{G}_{c}.$$

Entering the expression of shear modulus ${G}_{c}={E}_{c}/\left[2\left(1+\nu \right)\right]$ in Eq. (9), it becomes

10 $$\tau \left(x\right)=\frac{{u}_{s}(x)}{c}\frac{{E}_{c}}{2(1+\nu )}.$$

where $\nu$ is the Poisson’s ratio taken 0.2 for concrete.

**Figure 2:**
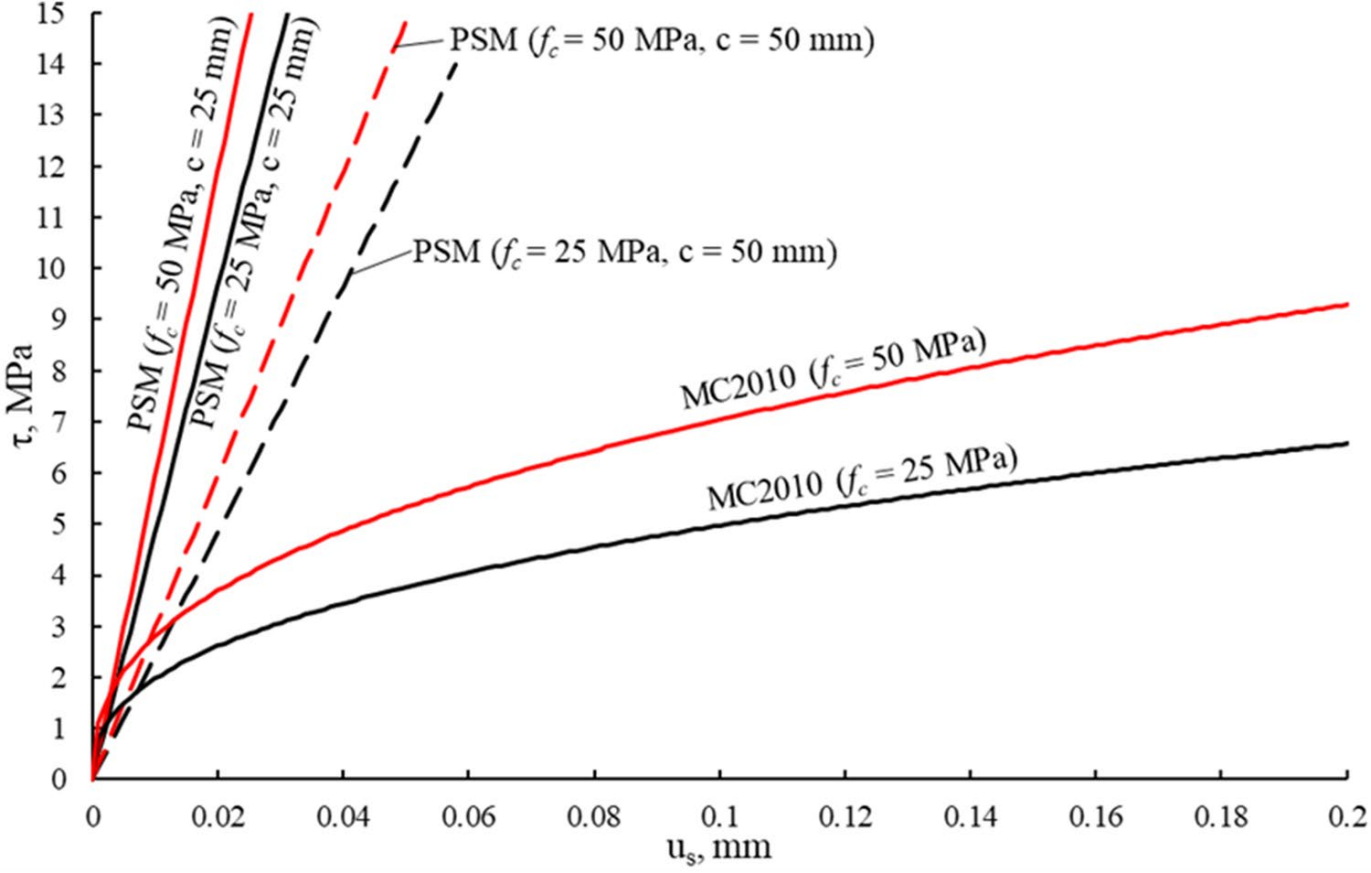
Bond–slip relations for the proposed model compared to the Model Code 2010^3^.

Equations (9) and (10) represent linear bond–slip law $\tau \left(x\right)=k{u}_{s}\left(x\right)$ with bond stiffness taken as $k={G}_{c}/c$. Figure 2 depicts bond–slip relations for different cover and concrete grade values compared to the ascending branch of the law given in the Model Code 2010^3^. It can be seen from Fig. 2 that in the early loading stages, the two models exhibit a rather similar bond stiffness, but at larger loads, the PSM demonstrates a significantly stiffer response. It should be noted that the two methods treat slip in a slightly different manner. While Model Code 2010^3^ assumes that slip is a difference between the displacements of reinforcement and concrete, the PSM model ignores concrete axial strains. However, the difference between the approaches is insignificant due to small concrete strains.

Entering the above $\tau \left(x\right)$ expression in Eq. (7) yields to

11 $$\frac{{d}^{2}{u}_{s}(x)}{d{x}^{2}}=\frac{2{E}_{c}}{{E}_{s}{d}_{b}c\left(1+\nu \right)}{u}_{s}\left(x\right).$$

Making a substitution:

12 $$\mathrm{\rm K}=\frac{2{E}_{c}}{{E}_{s}{d}_{b}c\left(1+\nu \right)}=\frac{2}{{nd}_{b}c(1+\nu )}$$

and rearranging Eq. (11):

13 $$\frac{{d}^{2}{u}_{s}(x)}{d{x}^{2}}-\mathrm{\rm K}{u}_{s}\left(x\right)=0,$$

where $n={E}_{s}/{E}_{c}$ is the modular ratio.

Equation (13) is a second order homogenous differential equation having this general solution:

14 $${u}_{s}\left(x\right)={C}_{1}{e}^{x\sqrt{\mathrm{\rm K}}}+{C}_{2}{e}^{-x\sqrt{\mathrm{\rm K}}}.$$

The integration constants ${C}_{1}$ and ${C}_{2}$ are determined from the boundary conditions using Eq. (5) and Fig. 1

15 $${u}_{s}\left(0\right)=0 \frac{d{u}_{s}}{dx}\left(\frac{{s}_{r}}{2}\right)={\varepsilon }_{s}\left(\frac{{s}_{r}}{2}\right)=\frac{P}{{E}_{s}{A}_{s}}={\varepsilon }_{si},$$

where ${\varepsilon }_{si}$ is the reinforcement strain in the cracked section. For these boundary conditions, Eq. (14) gets the shape:

16 $${u}_{s}\left(x\right)=\frac{{\varepsilon }_{si}}{\left(1+{e}^{{s}_{r}\sqrt{\mathrm{\rm K}}}\right)\sqrt{\mathrm{\rm K}}}\left({e}^{(x+0.5{s}_{r})\sqrt{\mathrm{\rm K}}}-{e}^{\left(0.5{s}_{r}-x\right)\sqrt{\mathrm{\rm K}}}\right).$$

Reinforcement strain,${\varepsilon }_{s}\left(x\right)$, can be expressed from Eqs. (5) and (16):

17 $${\varepsilon }_{s}\left(x\right)=\frac{d{u}_{s}\left(x\right)}{dx}=\frac{{\varepsilon }_{si}}{1+{e}^{{s}_{r}\sqrt{\mathrm{\rm K}}}}\left({e}^{(x+0.5{s}_{r})\sqrt{\mathrm{\rm K}}}+{e}^{\left(0.5{s}_{r}-x\right)\sqrt{\mathrm{\rm K}}}\right).$$

Assuming the mean value for crack spacing, ${s}_{r}={s}_{rm}$, Eq. (16) gets the shape

18 $${u}_{s}\left(x\right)=\frac{{\varepsilon }_{si}}{\left(1+{e}^{{s}_{rm}\sqrt{\mathrm{\rm K}}}\right)\sqrt{\mathrm{\rm K}}}\left({e}^{(x+0.5{s}_{rm})\sqrt{\mathrm{\rm K}}}-{e}^{\left(0.5{s}_{rm}-x\right)\sqrt{\mathrm{\rm K}}}\right).$$

Further modification involving Eqs. (4) and (18) results in this mean crack width expression:

19 $${w}_{m}=\frac{2{\varepsilon }_{si}}{\sqrt{\mathrm{\rm K}}}\frac{\left({e}^{{s}_{rm}\sqrt{\mathrm{\rm K}}}-1\right)}{\left({e}^{{s}_{rm}\sqrt{\mathrm{\rm K}}}+1\right)}.$$

**Figure 3:**
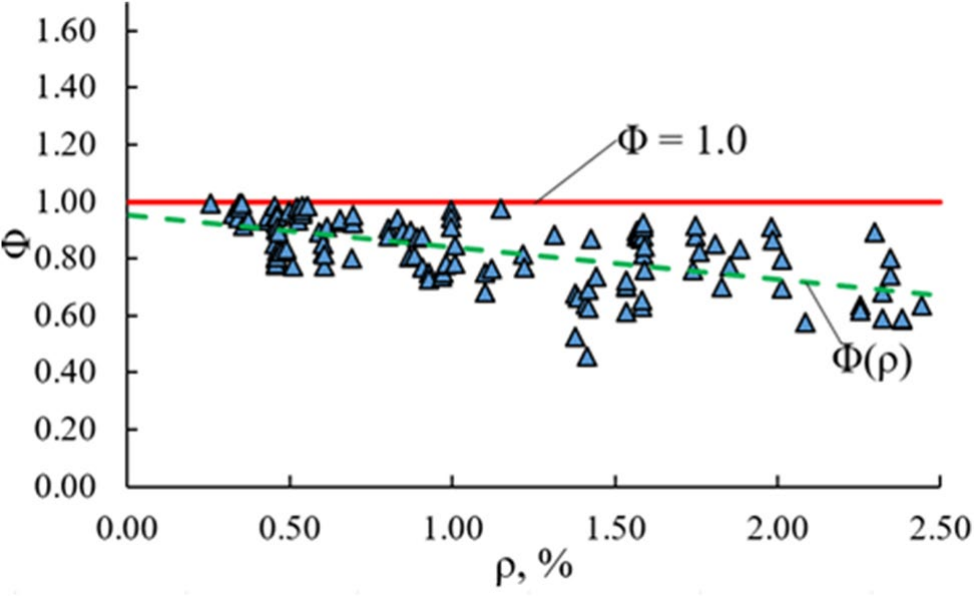
Parameter $\Phi =\frac{\left({e}^{{s}_{rm}\sqrt{\rm K}}-1\right)}{\left({e}^{{s}_{rm}\sqrt{\rm K}}+1\right)}$ versus reinforcement ratio, $\rho$.

Mean crack spacing, ${s}_{rm}$, remains the only unknown parameter in the calculation of ${w}_{m}$. As the prediction of ${s}_{rm}$ complicates the crack width analysis, further modifications of Eq. (19) are proposed to eliminate ${s}_{rm}.$ Mathematically the term $\frac{{\left( {e^{{s_{rm} \sqrt {\rm K} }} - 1} \right)}}{{\left( {e^{{s_{rm} \sqrt {\rm K} }} + 1} \right)}} = \Phi$ cannot exceed 1.0, and for most RC ties and bending members gets a value close to 1.0. The term clearly depends on ${s}_{rm}$ that in turn is related to the reinforcement ratio, $\rho$. The graph of $\Phi$ versus $\rho$ is depicted in Fig. 3 which uses test data of 160 RC beams described in^34–39^. While the mean value of $\Phi$ for the given test data is 0.82, the Pure Shear Model assumes $\Phi = 1.0$ what makes the results conservative and the mean crack width formula simpler:

20 $${w}_{m}=\frac{2{\varepsilon }_{si}}{\sqrt{\mathrm{\rm K}}}={\varepsilon }_{si}\sqrt{2{nd}_{b}c(1+\nu )}$$

In Eq. (20), ${w}_{m}$ is linearly related to the reinforcement strain of a fully cracked section. To a lesser extent, ${w}_{m}$ is dependent on the modular ratio, bar diameter, and cover thickness.

Maximum crack width, ${w}_{max}$, can be calculated by relating it to the mean crack width as suggested by CEB and Braam^40,41^:

21 $${w}_{max}=\beta {w}_{m}=1.7{w}_{m}.$$

## Comparison of crack width predictions to tests

This section compares mean crack width, ${w}_{m}$, predictions by the proposed model against the test data of RC ties and beams reported in the literature. The comparison also includes the predictions by Eurocode 2^2^ and Model Code 2010^3^.

### Tension RC members

The analysis employs two test programs^27,42^. All the members were singly reinforced with deformed bars and had essentially the same nominal geometry: a square section of 100 × 100 mm and a length of 1100 or 1150 mm. Tension on RC ties was exerted by pulling the ends of reinforcement.

Main geometrical and material parameters of the ties, such as section height, $h$, width, $b$, length, $L$, cylinder strength, ${f}_{cm}$, modulus of elasticity of concrete, ${E}_{c}$, and reinforcement, ${E}_{s}$, bar diameter, ${d}_{b}$, concrete cover, $c$, reinforcement ratio, $\rho$, and free shrinkage strain, ${\varepsilon }_{shr}$, are presented in Table 1.

**Table 1:** Main characteristics of test RC ties.

No	Name	Authors	h × b × L (mm_	f_cm_ (MPa)	E_c_ (MPa)	E_s_ (MPa)	d_b_ (mm)	c (mm)	ρ (%)	ε_shr_, × 10^−6^
1	STN12	Wu and Gilbert^27^	100 × 100 × 1100	21.56	22,400	200,000	12	44	1.13	− 28
2	STN16	Wu and Gilbert^27^	100 × 100 × 1100	21.56	22,400	200,000	16	42	2.01	− 28
3	STS12	Wu and Gilbert^27^	100 × 100 × 1100	24.73	21,600	200,000	12	44	1.13	− 249
4	STS16	Wu and Gilbert^27^	100 × 100 × 1100	24.73	21,600	200,000	16	42	2.01	− 249
5.–125.	N10-1-1-H52-20-3	Farra and Jaccoud^42^	100 × 100 × 1150	29.90–87.10	27,000–41,800	200,000	10–20	40–45	0.79–3.14	–

The test program of Farra and Jaccoud^42^, consisting of 121 specimens, was designed to investigate the effect of reinforcement ratio, bar diameter, and concrete grade on crack spacing and width. These parameters, particularly concrete compressive strength, and reinforcement ratio, ranged within wide limits (Table 1). The test program of Wu and Gilbert^27^, including four specimens, considered the effects of bar diameter (12 and 16 mm) and shrinkage (occurring prior to loading) on crack width.

The results of the accuracy analysis that included 125 RC ties are presented in Table 2. Accuracy was judged by two parameters: the mean value represents consistency, and the coefficient of variation quantifies the scatter. The predictions were expressed in terms of the normalized mean crack width, ${w}_{m,pred}/{w}_{m,test}$, where ${w}_{m,pred}$ and ${w}_{m,test}$ are the predicted and test crack width, respectively. The predicted and experimental crack widths were taken at the reinforcement stress ${\sigma }_{s}=250 MPa$ assumed to represent the service load. As for some of the test specimens^42^, the maximum reinforcement stress was below 250 MPa, or the stress at first cracking was above 250 MPa, the ${\sigma }_{s}$ values closest to 250 MPa were taken. For these members, stress, ${\sigma }_{s}$, ranged from 187 to 330 MPa. Two specimens were excluded from the original tests of Farra and Jaccoud^42^ as ${\sigma }_{s}$ exceeded 400 MPa at the formation of the first crack.

**Table 2:** The prediction accuracy of mean crack width of RC ties.

			Test	EC2	MC2010	PSM	EC2	MC2010	PSM
No.	Author	σ_s_ (MPa)	w_m,test_ (mm)	w_m,EC2_ (mm)	w_m,MC2010_ (mm)	w_m,PSM_ (mm)	w_m,EC2_/w_m,test_	w_m,MC2010_/w_m,test_	w_m,PSM_/w_m,test_
1.–4.	Wu and Gilbert^27^	250	0.133–0.193	0.172–0.190	0.130–0.142	0.133–0.153	0.943–1.432	0.707–1.074	0.703–1.041
5.–125.	Farra and Jaccoud^42^	187–330	0.070–0.207	0.093–0.215	0.057–0.135	0.090–0.140	0.646–2.431	0.458–1.373	0.506–1.551
						Avg.	1.139	0.762	0.886
						COV	0.335	0.232	0.207

As seen from Table 2, regarding scatter, the proposed model has demonstrated superior accuracy regarding the design code methods. For the PSM, the coefficient of variation was 21% compared to 23 and 34% as obtained for Model Code 2010 and Eurocode 2, respectively. However, the mean value for the PSM was not safe, with the prediction being, on average 11%, below the test. Model Code 2010 predictions were also unsafe (− 24%), whereas only Eurocode 2 gave safe predictions (+ 14).

**Figure 4:**
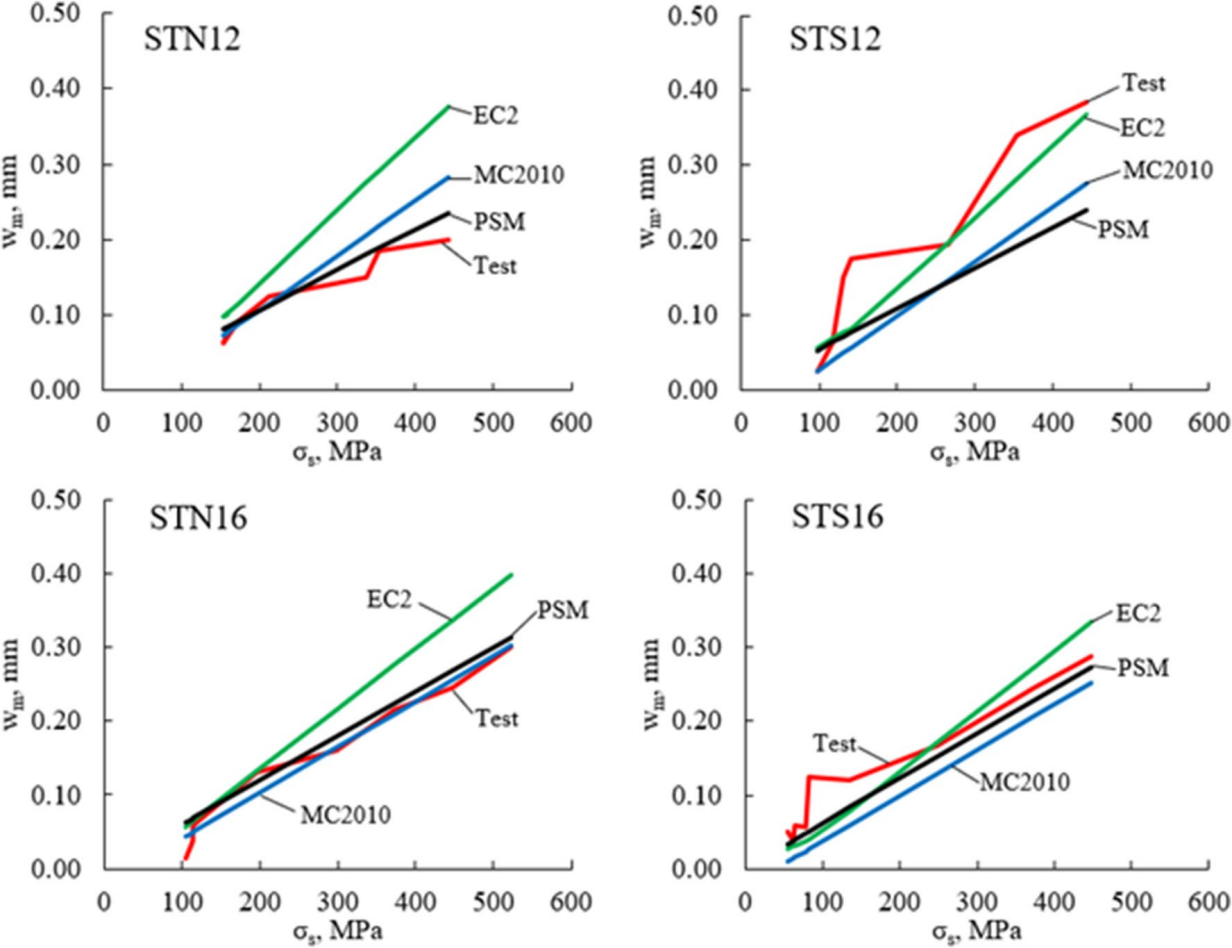
Mean crack width, ${\mathrm{w}}_{\mathrm{m}}$, predictions for test RC ties^27^.

The tests by Wu and Gilbert^27^ have given an opportunity to consider in more detail the shrinkage effect on short–term crack width. As seen from Table 1, the test ties from^27^ differed in bar diameter (12 or 16 mm) and free shrinkage strain, ${\varepsilon }_{shr}$. The members designated as STN were kept in humid conditions and had very little shrinkage, whereas STS members were kept in drying conditions and had ${\varepsilon }_{shr}$ about 9 times larger than STN members. The test and predicted ${w}_{m}$–${\sigma }_{s}$ graphs are depicted in Fig. 4 for each specimen. As expected, for both bar diameters, STS specimens had larger crack widths than STN members. The proposed model accurately predicted crack width for STN members and less so for STS specimens, particularly STS12.

The proposed model has the potential to be improved by considering the shrinkage effect. For that, a more advanced approach can be used based on Eq. (2) which includes the displacement components of shear lag, ${u}_{shear}$, and shrinkage, ${u}_{shr}$. A statistical analysis using the test data of Table 1, resulted in the mean value that approached unity. However, the analysis becomes more complex and requires the knowledge of ${s}_{rm}$ that, in this case, was assumed from the tests.

### Bending RC members

While the current model was derived for tension RC members, it can be applied for bending members as well. A limited comparative analysis of the predictions to the tests, presented below, includes six beams taken from two test programs^35,38^. As shown in Table 3, the beams differed in height, reinforcement ratio, bar diameter, and concrete grade. Four beams with section height $h=0.625 m$ were taken from the well–known tests of Rüsch and Rehm^35^, and two beams with $h=0.300 \mathrm{m}$ were employed from the experimental program of the authors^38^. All the beams were reinforced with deformed bars having a nominally equal bottom and lateral cover thickness. The tests were performed under a four–point bending scheme with mean crack width ${w}_{m}$ defined for the cracks within the pure bending zone.

**Table 3:** Main characteristics of RC beams.

No	Name	h × b × L (mm)	f_cm_ (MPa)	E_c_ (MPa)	E_s_ (MPa)	d_b_ (mm)	c (mm)	ρ (%)	ε_sh_, × 10^−6^
1	R26^35^	625 × 300 × 4500	14.35	24,515*	200,000	16	35	0.46	–
2	R70^35^	625 × 300 × 4500	14.18	24,429*	200,000	26	25	0.60	–
3	R14^35^	625 × 300 × 4500	13.84	24,253*	200,000	16	30	0.46	–
4	R69^35^	625 × 300 × 4500	13.50	24,074*	200,000	26	26	0.91	–
5	S200-1.98-10^38^	200 × 200 × 2500	50.90	27,661	200,000	10	24	1.96	− 390
6	S200-1.76-10^38^	200 × 200 × 2500	50.90	27,661	200,000	20	29	1.81	− 390

*Determined using Eurocode 2^2^.

**Figure 5:**
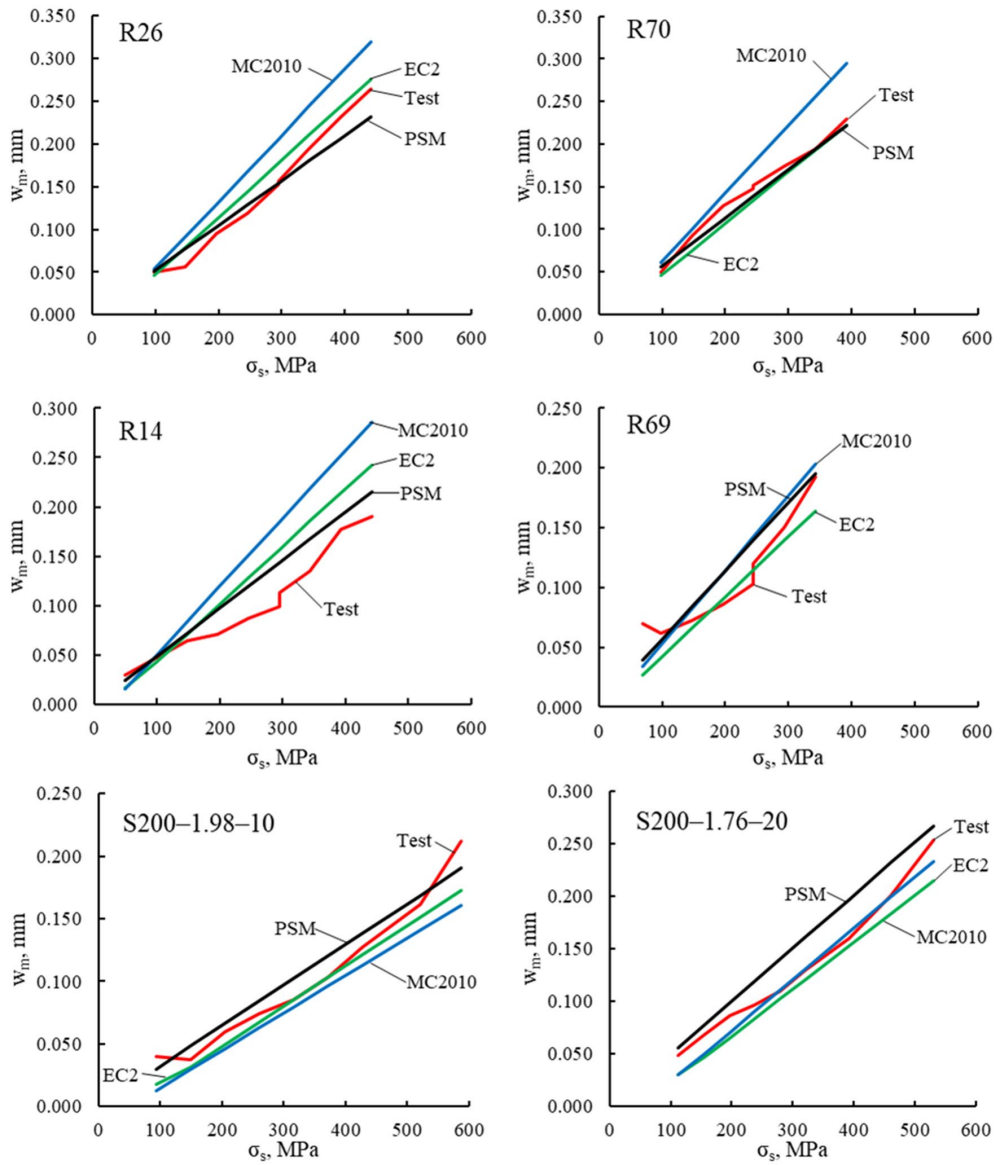
Mean crack width, ${w}_{m}$, versus reinforcement stress, ${\sigma }_{s}$.

The ${w}_{m}$–${\sigma }_{s}$ graphs for each of the beams are shown in Fig. 5. Numerical ${w}_{m}$ values at ${\sigma }_{s}=250 \mathrm{MPa}$ are presented in Table 4. In general, all three models quite accurately predicted mean crack widths. Compared to the other two models, the proposed model produced the smallest scatter. However, due to a small number of the test specimens, no generalized conclusions can be drawn about the prediction accuracy of the models.

**Table 4:** Mean crack width predictions for test RC beams.

			Test	EC2	MC2010	PSM	EC2	MC2010	PSM
No	Name	σ_s_ (MPa)	w_m,test_ (mm)	w_m,EC2_ (mm)	w_m,MC2010_ (mm)	w_m,PSM_ (mm)	w_m,EC2_/w_m,test_	w_m,MC2010_/w31_test_	w_m,PSM_/w_m,test_
1	R26^35^	250	0.123	0.148	0.171	0.131	1.205	1.395	1.067
2	R70^35^		0.154	0.137	0.182	0.141	0.890	1.180	0.918
3	R14^35^		0.088	0.131	0.154	0.312	1.484	1.747	1.380
4	R69^35^		0.123	0.117	0.146	0.142	0.953	1.184	1.157
5	S200-1.98-10^38^		0.072	0.064	0.060	0.081	0.893	0.832	1.127
6	S200-1.76-10^38^		0.101	0.089	0.097	0.126	0.886	0.963	1.249
						Avg	1.052	1.217	1.150
						COV	0.232	0.267	0.137

## Concluding remarks

1. The study proposes a new analytical model for the mean crack width analysis of RC members reinforced with ribbed bars and subjected to short–term load.
2. The proposed approach is based on the partial interaction tension stiffening model considering a short RC tie with elastic material properties. The model is derived by equating the longitudinal displacements of reinforcement and concrete. For simplicity, slip between concrete and reinforcement at their interface as well as the internal cracking of concrete and concrete strains due to tension stiffening and shrinkage are ignored.
3. The only deformations assumed in concrete are the shear strains due to shear lag that are taken constant across the cover thickness. Deplanation of concrete section due to shear lag results in crack width linearly increasing from zero at the bar to the maximum value on the surface of the RC member.
4. The proposed approach is named the Pure Shear Crack Model in relation to the assumption of constant shear strain. The model predicts the surface mean crack width by a simple formula expressed via reinforcement strain, bar diameter, concrete cover, modular ratio, and Poisson’s ratio for concrete.
5. The proposed model combines the features of two main concepts in crack width analysis. Similar to the no–slip approach, the model neglects slip and takes into account the section deplanation due to shear lag. Likewise, the stress–transfer approach, PSM is capable of transferring stress from reinforcement to concrete via the bond stress.
6. Despite the analytical background and simplicity of the proposed model, its accuracy in predicting mean crack width in tension and bending RC members was shown to be comparable to that of fashionable design codes. For an extensive data set of RC ties (125 specimens), the coefficient of variation was 21% compared to 23% and 34% for Model Code 2010 and Eurocode 2, respectively. However, the mean value for the PSM was not safe, with the prediction being 11% below the test. The latter outcome could be improved by including the shrinkage effect. The predictions by Model Code 2010 were also unsafe (–24%), whereas Eurocode 2 gave safe predictions (+ 14%).

## Data Availability

The datasets used and analyzed during the current study are available from the corresponding author on reasonable request.
